# High-Intensity Inspiratory Protocol Increases Heart Rate Variability in Myocardial Revascularization Patients

**DOI:** 10.5935/1678-9741.20160007

**Published:** 2016

**Authors:** Flavia Cristina Rossi Caruso, Rodrigo Polaquini Simões, Michel Silva Reis, Solange Guizilini, Vera Lucia dos Santos Alves, Valeria Papa, Ross Arena, Audrey Borghi-Silva

**Affiliations:** 1 Laboratory of Cardiopulmonary Physiotherapy at Federal University of São Carlos (UFSCar), São Carlos, SP, Brazil.; 2 Department of Physiotherapy at Faculty of Medicine at Federal University of Rio de Janeiro (FMUFRJ), Rio de Janeiro, RJ, Brazil.; 3 Department of Sciences of Human Movement at Federal University of São Paulo (UNIFESP), Santos, SP, Brazil.; 4 Faculty of Medical Sciences at Santa Casa de São Paulo (FCMSCSP), São Paulo, SP, Brazil.; 5 Hospital São Francisco of Ribeirão Preto (SF), Ribeirão Preto, SP, Brazil.; 6 Department of Physical Therapy and Integrative Physiology Laboratory, College of Applied Health Sciences, University of Illinois Chicago, Chicago, IL, USA.

**Keywords:** Autonomic Nervous System, Respiratory Muscles, Heart Rate, Physical Therapy Modalities, Coronary Artery Bypass

## Abstract

**Objective:**

To evaluate heart rate variability during an inspiratory muscle endurance protocol at three different load levels [30%, 60% and 80% of maximal inspiratory pressure], in patients who had previously undergone coronary artery bypass grafting.

**Methods:**

Nineteen late postoperative myocardial revascularization patients participating in a cardiovascular rehabilitation program were studied. Maximal inspiratory pressure maneuvers were performed. An inspiratory muscle endurance protocol at 30%, 60% and 80% of maximal inspiratory pressure was applied for four minutes each, in random order. Heart rate and RR intervals were recorded and heart rate variability was analyzed by time (RMSSD-the mean of the standard deviations for all R-R intervals, and RMSM-root-mean square differences of successive R-R intervals) and frequency domains indices (high and low frequency) in normalized units. ANOVA for repeated measurements was used to compare heart rate variability indices and Student t-test was used to compare the maximal inspiratory pressure and maximal expiratory pressure values.

**Results:**

Heart rate increased during performance of maximal respiratory pressures maneuvers, and the maximal inspiratory pressure and maximal expiratory pressure mean values were significantly lower than predicted values (*P*<0.05). RMSSD increased significantly at 80% in relation to rest and 30% of maximal inspiratory pressure and RMSM decreased at 30% and 60% of maximal inspiratory pressure in relation to rest (*P*<0.05). Additionally, there was significant and progressive decrease in low frequency and increase in high frequency at 30%, 60% and 80% of maximal inspiratory pressure in relation to the resting condition.

**Conclusion:**

These results suggest that respiratory muscle training at high intensities can promote greater parasympathetic activity and it may confer important benefits during a rehabilitation program in post-coronary artery bypass grafting.

**Table:** 

**Abbreviations, acronyms & symbols**	
ANOVA	= Analysis of variance
BMI	= Body mass index
CABG	= Coronary artery bypass grafting
CAD	= Coronary artery disease
COPD	= Chronic obstructive pulmonary disease
ECG	= Electrocardiogram
HFun	= High frequency in normalized units
HR	= Heart rate
HRV	= Heart rate variability
LFnu	= Low frequency in normalized units

MEP	= Maximal expiratory pressure
MIP	= Maximal inspiratory pressure
MRP	= Maximal respiratory pressure
RMS	= Respiratory muscle strength
RMSM	= Root-mean square differences of successive R-R intervals
RMSSD	= Mean of the standard deviations for all R-R intervals
RMT	= Respiratory muscle training
RSA	= Respiratory sinus arrhythmia
RV	= Residual volume
TPC	= Total pulmonary capacity

## INTRODUCTION

The evaluation of autonomic cardiac function by heart rate variability (HRV) in patients with coronary artery disease (CAD) has been widely used for risk stratification^[1-3]^. Studies have been developed to evaluate HRV in patients with CAD who have undergone coronary artery bypass grafting (CABG) surgery^[4,5]^; the benefits being HRV assessment is non-invasive, low cost and predicts cardiovascular morbidity and mortality in early and late phases of surgery procedure^[2,6,7]^.

Respiratory muscle strength (RMS) evaluation by maximal respiratory pressure (MRP) is used as an important diagnostic and prognostic measure in patients with neuromuscular^[8]^, pulmonary^[9]^ and cardiovascular disease^[10]^. Moreover, respiratory muscle training (RMT) have proven to be a valuable treatment approach in preventing pulmonary complications after CABG^[11]^. Collectively, the simultaneous evaluation of autonomic cardiac function and RMS may play an important role in the evaluation of cardiorespiratory integrity in patients following CABG^[12]^.

CABG produces an important negative impact on autonomic cardiac function^[13,14]^ and RMS^[15]^. Previous studies have reported that return of RMS takes several months following CABG^[16]^. Participation in cardiac rehabilitation induces a host of benefits, including improved cardiac autonomic function^[17]^ and RMS^[18]^.

To our knowledge, no previous study has assessed the cardiac autonomic system by HRV during different loads resistance loads imposed upon the respiratory musculature in patients post-CABG; this has important implications for establishing RMT intensities that optimally improve HRV^[19-21]^. Therefore, the aim of this study was to assess HRV during an inspiratory muscle endurance protocol at three different levels of effort [30%, 60% and 80% of maximal inspiratory pressure (MIP)] in patients post-CABG. We hypothesized that the application of a high-intensity inspiratory muscle endurance protocol may induce greater changes in HRV when contrasted to moderate and low loads.

## METHODS

### Subjects

Twenty eight patients who had previously undergone CABG were recruited to participate in the present study and 19 male patients satisfied all inclusion criteria. All subjects participated in a cardiovascular physical therapy program [60 minute sessions three times a week for at least 6 months at 70-85% of maximum heart rate (HR)]. Exclusion criteria were emergent or concomitant surgery, implanted pacemaker, unstable angina, recent myocardial infarction (less than 6 months), chronic disturbances in heart rhythm that could compromise HRV analysis, chronic obstructive pulmonary disease (COPD), valvular heart disease, severe non-cardiac diseases, and the inability to perform the study protocol. Patients who were obese [body mass index (BMI) > 30 kg/m^2^], active smokers, had evidence of left ventricular dysfunction, neurological and respiratory disturbances, visible alterations in thoracic and/or abdominal mobility, or accentuated structural deviations in the spine that might alter the respiratory dynamic were excluded.

Time from completion of surgery to the entry in the study protocol was 180±12 days. All subjects were oriented to the experimental procedures to be performed and they signed a written informed consent agreement in accordance with resolution 196/96 of the National Health Council. This study was reviewed and approved by the Ethics Committee for Human Research (number 109/2006).

### Experimental Design

The study procedures were performed in the cardiopulmonary laboratory of our institution, in the morning to avoid any circadian variations, with a room temperature controlled at 22 to 24°C and at a relative air humidity of 50% to 60%.

The patients were instructed to not ingest alcohol or other stimulants the night before and day of the experimental procedures, to not do any heavy physical exercise, to avoid heavy meals for two hours before the experimental procedures and to get a good night's sleep the night before.

### Clinical Evaluation

All patients underwent clinical evaluation which consisted of: 1) anamneses; 2) past medical and surgical history; 3) family medical history; 4) risk factor profile; 5) lifestyle habits; 6) visual inspection to identify possible alterations in the thoracic and abdominal regions such as cutaneous folds and accentuated structural deviations in the spine that might alter respiratory dynamics; 7) anthropometric evaluation measuring height and body mass by stadiometer and scale (Welmy, São Paulo, SP, Brazil); 8) 12-lead standard electrocardiogram (ECG) measurement of HR (cardiac monitor - Ecafix TC 500, São Paulo, SP, Brazil); 9) arterial blood pressure (sphygmomanometer BD, São Paulo, SP, Brazil); 10) maximum dynamic physical effort test; and 11) laboratory exams (fasting glycemia, total and fractions cholesterol, triglycerides, uric acid, creatine and type 1 urine).

### Respiratory Muscle Strength

To obtain values for MIP and maximal expiratory pressure (MEP), an aneroid type manovacuometer (GER-AR, São Paulo, SP, Brazil) with an operational interval of ± 300 cmH_2_O was used. A plastic mouthpiece was coupled with a tube attached to the manovacuometer. This mouthpiece had a leak^[22]^ with a diameter of approximately 2 mm that permitted a small amount of air to escape to avoid any elevation of pressure within the oral cavity by contraction of the facial muscles^[23]^. Each individual used a rubber mouthpiece with a diameter of 32 mm over the plastic mouthpiece.

Before the measurements were taken, patients remained seated while being familiarized with the equipment and instructed on how to perform the maneuvers. Immediately following, the HR and R-R intervals were registered for three periods: 1) during the first minute at rest; 2) for approximately three seconds of the maneuver; and 3) during the final minute of the procedure.

The MIP and MEP maneuvers were performed a minimum of three times with two minute intervals between repetitions. During rest periods (first and last) between each maneuver, the patient was oriented to remain seated, quiet and still to avoid interference with the ECG signal being recorded. Ten seconds before the maneuver was performed, a nasal clip was placed on the patient (to not allow air to escape from the nostrils), and the patient was instructed to keep their lips tightly closed over the mouth while performing the forced inspiration maneuver from residual volume (RV) and the forced expiration maneuver from total pulmonary capacity (TPC) to maintain maximum respiratory effort for approximately one second^[23]^.

The greatest values obtained from three correctly performed repetitions (with a 10% or less difference between values) for each maneuver were registered. It is important to emphasize that a single evaluator performed the manovacuometry for all individuals and verbal encouragement was given to all patients during the maneuvers to reach the maximal effort of the patient. The MIP and MEP measurement were compared to the predicted values for Brazilian population according to Simões et al.^[23]^.

### Inspiratory Muscle Endurance Protocol

The inspiratory muscle endurance protocol consisted of maneuvers at three pressure levels: 30%, 60% and 80% of MIP. The loads were applied in a random order by drawing of shuffled, opaque, coded envelopes that were opened by one investigator. First, the subjects were maintained at rest for 10 minutes and the HR data was obtained while subjects rested quietly, breathing spontaneously in the seated position.

During the protocol, the patient remained seated in a chair, using a nose clip and performed inspiratory efforts using the manovacuometer which had previously shown to the value that corresponded to the individual's pressure percentage of MIP (30%, 60% or 80%). Each effort level was performed for four minutes and the patient was oriented to make an inspiratory effort and maintain the equipment indicator on the demarcated line, which corresponded to the percentage being tested, for two seconds followed by expiration through the mouth for three seconds; this corresponded to a total of 12 respiratory cycles per minute^[24]^. To ensure that the maneuver was performed correctly and at the correct times for inspiration and expiration as previously instructed, one of the evaluators used a chronometer to give verbal commands to the patient^[24]^.

The HR was continually registered one minute before the beginning of effort, during four minutes at each effort of intensity, and during the first minute after the effort. During this period, the ECG signal and HR were observed in real time on the computer monitor to verify signal quality.

### Heart Rate and R-R Intervals Data Collection

The ECG signal, R-R intervals and the HR were obtained on a beat to beat basis in real time through a cardiac monitor registering the derivation of the CM5 lead, using disposable self adhesive activated carbon electrodes (red electrode positioned on the esternal manubrium, yellow electrode in the 5^th^ left intercostals space on the anterior axillary line, and black electrode in the 5^th^ right intercostals space).

The ECG signal was transferred to a microcomputer (PC-AT 486 DX-4, 100MHz) through an analog-digital converter (Lab. PC + National Instruments, Co.), that constituted an interface between the cardiac monitor and the microcomputer. The analog signals taken from the ECG were converted into binary values and processed by software specifically designed to capture ECG and calculate the R-R intervals^[25]^.

### Data Analysis

The HRV was analyzed by the time and frequency domain methods. In the time domain, the R-R intervals were analyzed by the root-mean square differences of successive R-R intervals (RMSM) and mean of the standard deviations for all R-R intervals (RMSSD) indices. The RMSM index corresponded to the square root of the sum of the square of the differences of the individual values in relation to the mean value, divided by the number of R-R intervals in a specified time period^[26]^. The RMSSD corresponded to the square root of the sum of the square of the differences between the R-R intervals registered, divided by the number of R-R intervals in a specified time period less one^[26]^. To the frequency domain analysis, 256 points of the four minutes (endurance protocol) was selected as the criterion required for application of the spectral methods (i.e., Fast Fourier Transforming). Then, the power spectral components were obtained at low and high frequencies in normalized units [low frequency in normalized units (LFnu) and high frequency in normalized units (HFnu), respectively].

### Statistical Analysis

On the basis of the results of the pilot study (n=8), we estimated that a sample size of 15 individuals would have a power >80% to detect a 5 ms difference in the RMSSD index (main outcome) amongst the imposed inspiratory loads. The level of significance was set at 5%. The data distribution was verified by the Shapiro-Wilk test, and when normality was confirmed the data were expressed in mean and standard deviation. Analysis of variance (ANOVA) for repeated measurements was used to compare the indexes of HRV (RMSM, RMSSD, LFnu and HFnu) obtained during the endurance protocol at four different situations: rest (pre-effort), 30%, 60% and 80% of MIP. The Student *t*-test for dependent samples was used to compare the MIP and MEP values obtained by the manovacuometer to the predicted values^[23]^ and to compare the HR and R-R intervals values obtained during rest (pre-effort) with peak values achieved during MIP. The probability of a type I error was established at 5% for all tests (α=0.05). The data was analyzed using the STATISTICA for Windows software program (Stat Soft Inc, 2000).

## RESULTS

During the recruitment 11 patients were excluded due to follow reasons: 1) 3 patients presented complexes arrhythmias; 2) 2 patients underwent concomitant CABG and valvular surgery; 3) 1 patient had a COPD diagnosis; 4) 3 patients presented with left ventricle dysfunction; and 5) 2 had difficulty comprehending the protocol due to previous stroke. All 19 participants included in the study completed the endurance protocols and had no clinically relevant adverse events throughout the study. All participants underwent elective CABG with cardiopulmonary bypass, median sternotomy incision and interposition of a saphenous vein or radial artery graft. All participants received internal mammary artery grafting. None of the subjects presented with pulmonary or hemodynamic complications in the perioperative period. Mean of cardiopulmonary bypass time was 78±22 min, time of surgery procedure was 178±60 min, time to mechanical ventilation was 10±5.8 h, intensive care unit stay was 2.3±1.2 days and total time in the hospital was 7±2 days. All patients underwent grafting with mammary artery.

Table 1 lists the anthropometric characteristics of the patients studied in relation to age, weight, height and BMI, as well as risk factors and number and type of grafts used in CABG. The medications taken by the patients during the evaluation period and the respective number of patients were: 1) acetyl salicylic acid (12 patients); 2) anti-arrhythmic (2 patients); 3) angiotensin converting enzyme (15 patients); 4) β-blockers (12 patients); 5) calcium-channel blockers (5 patients); 6) nitrates (7 patients); 7) angiotensin II receptor antagonists (2 patients); 8) antihypercolesterolemics (9 patients); and 9) diuretics (5 patients).

**Table 1:** Age, anthropometric characteristics, risk factors and number and type of grafts used in coronary artery bypass surgery.

Age and Anthropometric Characteristics	n=19
Age (years)	68±5
Weight (kg)	78±25
Height (m)	1.69±0.08
BMI (kg/m^2^)	27±4.2
Risk Factors	n
Hypertension	12
History of CAD	16
Dyslipidemia	11
Ex-smoker	13
Grafts	n (quantity and type)
4 grafts	4 (3v and 1a)
3 grafts	8 (2v and 1a)
2 grafts	5 (1v and 1a)
1 graft	2 (a)

Data expressed in mean and standard deviation. BMI=body mass index; n=number of patients; CAD=coronary arterial disease; v=venous graft; a=arterial graft.

Subjects presented with significantly lower MIP and MEP values (*P*<0.05) when compared to predicted values (Table 2). In relation to HR during the MIP and MEP maneuvers, a significant increase was found at peak when compared to rest. This fact can be confirmed by the reduction in R-R intervals during the MIP and MEP, in comparison to the pre-effort rest period (Table 2).

**Table 2:** Comparison of maximal respiratory pressures, heart rate and R-R intervals values under pre effort rest conditions and during maximal inspiratory and expiratory pressures maneuvers.

Variables	MIP (n=19)	MEP (n=19)
MRP obtained (cmH_2_O)	68±15	78±20
MRP predicted (cmH_2_O)	78±10*	75±12*
HR rest (bpm)	65±10	66±9
HR peak (bpm)	98±12†	102±10†
R-Ri rest (ms)	1012±220	1112±176
R-Ri peak (ms)	613±101†	615±180†

Data expressed in mean and standard deviation. MIP=maximal inspiratory pressure; MEP=maximal expiratory pressure; MRP=maximal respiratory pressure; HR=heart rate; R-Ri=R-R intervals

* P<0.05 between obtained and predicted values;

† P<0.05 between rest and peak conditions.

**Fig. 1:**
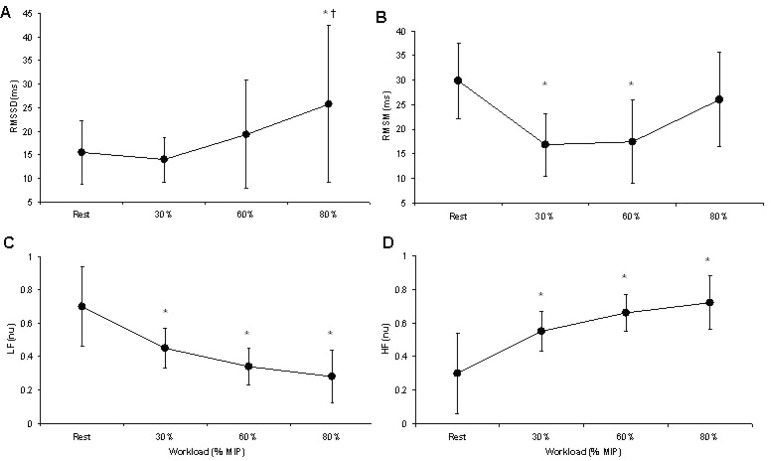
Comparison of heart rate variability index at rest, 30%, 60% and 80% of maximal inspiratory pressure during endurance protocol. Data expressed in mean and standard deviation. MIP=maximal inspiratory pressure; LFnu=low frequency in normalized units; HFun=high frequency in normalized units. *P<0.05 in relation to rest condition; †*P*<0.05 in relation to 30% of MIP.

The HRV evaluated during the endurance protocol at the different MIP percentages showed a significant increase in the RMSSD at 80% (Figure 1A) and significant decrease in the RMSM index at 30% and 60% of MIP in relation to rest (Figure 1B), and significant decrease and increase in the LFnu and HFnu, respectively, at 30%, 60% and 80% of MIP in relation to the resting condition (Figures 1C and 1D). However, only the 80% MIP maneuver demonstrated a significant increase in RMSSD and high frequency as well as reduced low frequency when contrasted with the 30% MIP maneuver.

## DISCUSSION

The main findings of the present study showed that after 6 months of CABG, reductions in MIP and MEP compared to predicted values were observed. In addition, we observed that the increase in inspiratory muscle workload (% of MIP) produced higher parasympathetic and lower sympathetic cardiac modulation when contrasted with moderate and low loads. These findings may have important implications for establishing RMT strategies following CABG.

Our results showed a significant increase in the RMSSD and HFnu index was observed (Figures 1A and 1D, respectively), indicating an increase in parasympathetic activity when the protocol was performed at a higher load. There was also a decrease in sympathetic activity with the increase of workload, as indicated by LFnu behavior (Figure 1C). In relation to the RMSSD, this index reflects both sympathetic and parasympathetic activity, therefore reduced values observed at 30% and 60% of MIP could be explained by a decrease in sympathetic modulation. Additionally, it was possible to observe an increase in the values of this index at 80% of MIP, this fact revealed a predominance of parasympathetic modulation at this load in the present study cohort.

A possible explanation for the increase in parasympathetic and decrease sympathetic modulation during a higher inspiratory workload is the increase in tidal volume, as well as the increase in inspiratory effort. In this context, some studies have demonstrated that the magnitude of autonomic responses of HR during respiratory sinus arrhythmia (RSA) maneuver is directly proportional to the tidal volume^[19]^.

The endurance protocol applied in the current study has a certain similarity to the RSA maneuver in which it was performed with controlled inspiratory and expiratory times. On the other hand, the protocol offers resistance to the inspiratory muscles. Also, a greater accentuation of the RSA is achieved because the maneuver performed at a higher frequency (i.e., 12 cycles per minute). However, we believe that the mechanisms involved in cardiac autonomic control with the respiratory maneuver are the same, and we base this on the probability of tidal volume having increased with the increase in workload, which in turn influenced the autonomic cardiac responses. Kautzner^[27]^ verified that there is evidence that the variations in tidal volume can potentially interfere with the reproducibility of RSA and in the capacity of the test to measure differences in vagal control.

Several experimental studies^[28,29]^ have shown that an increase in ventilation through an increase in tidal volume may cause fluctuations in autonomic cardiac modulation. For this reason, one speculates whether a RMT program in humans can influence total HRV. Laoutaris et al.^[30]^ verified in their study with chronic heart failure patients undergoing RMT at 60% of MIP that HR at rest was significantly reduced after 10 weeks of respiratory training (three times a week). According to these authors^[30]^, these findings may reflect a relation between the improvement in respiratory function and the changes in autonomic balance, favoring an increase in vagal activity. Another hypothesis is the relative attenuation of HR due to RMT would be related to the reflex mediated by the diaphragm muscle, that may influence the sympathetic tone; recent research^[31,32]^ has demonstrated that when muscle fatigue is induced in healthy individuals, a decrease in blood flow and increase in vascular resistance to the lower limbs is observed, resulting from sympathetic responses to diaphragmatic stress.

As to the MRP, the lower values for MIP as well as MEP in comparison to the predicted values indicate that the RMS in these patients is still reduced, suggesting that despite myocardial revascularization and participation in a cardiovascular rehabilitation program for 6 months, a decrease in respiratory muscle performance persists. Several studies^[33-37]^ have demonstrated that the reduction in RMS after myocardial revascularization surgery. These authors^[34-37]^ attribute this fact to the damage caused by mechanical respiration with thoracic incision, reducing the capacity of the respiratory muscles to generate sufficient tension to perform respiratory work imposed due to a mechanical disadvantage. Borghi-Silva et al.^[34]^ found that RMS suffers a significant reduction after CABG and that the pressure values verified on the day of hospital discharge remain lower than preoperative values. KristjAnsdottir et al.^[37]^ found that thoracic mobility is reduced even one year after surgical intervention. For this reason, we believe that these alterations in respiratory mechanics caused by thoracic incision could reduce the respiratory muscle efficiency of the patients evaluated. However, some authors^[36]^ have demonstrated that pulmonary function returns to normal values after six months of surgery.

In this context, an important aspect to note is that RMT has not been commonly employed in cardiovascular rehabilitation programs. However, based on the results of the present study, we believe that RMT should be applied not only in late phase post-CABG, but also in the immediate postoperative phase^[33]^ aiming at re-establishing RMS. In this context, RMT applied at higher intensities could be an important strategy to enhance vagal tone in parallel to aerobic exercise training programs in these patients. In particular, the positive effects of RMT on vagal tone could produce a cardioprotective effect, reducing risks to arrhythmias and fatal events.

Some limitations in this study should be taken into consideration. Although the patients were in a stable postoperative phase after surgical intervention, the sample was relatively small. However, the sample size to answer the main outcome was powered >80%. In addition, it was not possible to perform tidal volume measurement at the different percentages of MIP during the endurance protocol and it also was not possible to compare thoracic mobility during the protocol, aspects that would establish a more solid foundation for the findings observed in this study. Finally, the results of the present study are restricted to patients in the late phase of cardiac surgery. In this context, the impact of high intensity MIP maneuvers to produce a marked parasympathetic modulation during the early postoperative phase, when RMS is more profoundly impacted, requires further investigation.

## CONCLUSION

In conclusion, we found that after 6 months post-CABG reductions in MIP and MEP were persisted. In addition a high-intensity inspiratory protocol promoted a greater parasympathetic modulation in comparison to maneuvers at lower loads. These results provide important implications for rehabilitation procedures following CABG, in particular including a RMT component as a standard of care.

**Table:** 

**Authors' roles & responsibilities**	
FCRC	Analysis and/or data interpretation; statistical analysis; final manuscript approval
RPS	Conception and design study; realization of operations and/or trials; statistical analysis; final manuscript approval
MSR	Conception and design study; analysis and/or data interpretation; manuscript redaction or critical review of its content; final manuscript approval
SG	Analysis and/or data interpretation; manuscript redaction or critical review of its content; final manuscript approval
VLSA	Manuscript redaction or critical review of its content; final manuscript approval
VP	Analysis and/or data interpretation; manuscript redaction or critical review of its content; final manuscript approval
RA	Manuscript redaction or critical review of its content; final manuscript approval
ABS	Conception and design study; realization of operations and/or trials; statistical analysis; analysis and/or data interpretation; manuscript redaction or critical review of its content; final manuscript approval
